# Atenolol's Inferior Ability to Reduce Central vs Peripheral Blood Pressure Can Be Explained by the Combination of Its Heart Rate-Dependent and Heart Rate-Independent Effects

**DOI:** 10.1155/2020/4259187

**Published:** 2020-04-26

**Authors:** Tuuli Teeäär, Martin Serg, Kaido Paapstel, Mare Vähi, Jaak Kals, John R. Cockcroft, Mihkel Zilmer, Jaan Eha, Priit Kampus

**Affiliations:** ^1^Department of Cardiology, Institute of Clinical Medicine, University of Tartu, 8 Puusepa Street, Tartu 50406, Estonia; ^2^Heart Clinic, Tartu University Hospital, 8 Puusepa Street, Tartu 50406, Estonia; ^3^Centre of Cardiology, North Estonia Medical Centre, 19 Sütiste Street, Tallinn 13419, Estonia; ^4^Institute of Mathematics and Statistics, University of Tartu, 2 J. Liivi Street, Tartu 50409, Estonia; ^5^Department of Biochemistry, Centre of Excellence for Genomics and Translational Medicine, Institute of Biomedicine and Translational Medicine, University of Tartu, 19 Ravila Street, Tartu 50411, Estonia; ^6^Department of Surgery, Institute of Clinical Medicine, Tartu University Hospital, 8 Puusepa Street, Tartu 50406, Estonia; ^7^Surgery Clinic, Tartu University Hospital, 8 Puusepa Street, Tartu 50406, Estonia; ^8^Division of Cardiology, Department of Medicine, Columbia University, 622 West 168th Street, New York, NY 10032, USA

## Abstract

**Objective:**

Whether the inferior ability of atenolol to reduce central (aortic) compared to peripheral (brachial) blood pressure (BP) is related to its heart rate (HR)-dependent or -independent effects, or their combination, remains unclear. To provide further mechanistic insight into this topic, we studied the acute effects of atenolol versus nebivolol and ivabradine on systolic blood pressure amplification (SBPA; peripheral systolic BP minus central systolic BP) in a model of sick sinus syndrome patients with a permanent dual-chamber cardiac pacemaker in a nonrandomized single-blind single-group clinical trial.

**Methods:**

We determined hemodynamic indices noninvasively (Sphygmocor XCEL) before and at least 3 h after administration of oral atenolol 50 or 100 mg, nebivolol 5 mg, or ivabradine 5 or 7.5 mg during atrial pacing at a low (40 bpm), middle (60 bpm), and high (90 bpm) HR level in 25 participants (mean age 65.5 years, 12 men).

**Results:**

At the low HR level, i.e., when the drugs could exert their HR-dependent and HR-independent effects on central BP, only atenolol produced a significant decrease in SBPA (mean change 0.74 ± 1.58 mmHg (95% CI, 0.09–1.40; *P* = 0.028)), indicating inferior central vs peripheral systolic BP change. However, we observed no significant change in SBPA with atenolol at the middle and high HR levels, i.e., when HR-dependent mechanisms had been eliminated by pacing.

**Conclusion:**

The findings of our trial with a mechanistic approach to the topic imply that the inferior ability of atenolol to reduce central vs peripheral BP can be explained by the combination of its heart rate-dependent and -independent effects. This trial is registered with NCT03245996.

## 1. Introduction

One reason why *ß*-blockers (BB) are not recommended as first-line antihypertensives in uncomplicated arterial hypertension in guidelines with a global impact is their inferior ability to reduce cardiovascular events, especially stroke, compared to other antihypertensive drug classes [1–3]. Their weaker cardiovascular protection can partly be associated with less central blood pressure (BP) reduction compared to peripheral BP reduction [4–6]. Pucci et al. reported recently in a meta-analysis that the weaker ability of nonvasodilating BBs (NVBB, mainly atenolol) to reduce central versus peripheral systolic BP resulted primarily from HR reduction [7]. However, in our previous trial, we showed that a vasodilating BB nebivolol reduced central systolic BP contrary to metoprolol, a NVBB, despite a similar reduction in HR and peripheral systolic BP [8]. A trial with ivabradine, a HR-lowering drug without direct effects on myocardial contractility and vascular resistance, did not confirm the detrimental effect of pharmacological HR lowering on central BP, suggesting that HR-independent factors, such as peripheral vasoconstriction or myocardial contractility reduction, could explain the pseudo-antihypertensive effect of NVBBs [9].

To provide mechanistic insight into the question whether atenolol's inferior effect on central BP is associated with its HR-dependent or HR-independent properties, or their combination, we studied the acute effects of atenolol versus nebivolol and ivabradine on systolic BP amplification (SBPA; peripheral systolic BP minus central systolic BP) at different HR levels in a model of sick sinus syndrome patients with a permanent dual-chamber cardiac pacemaker.

## 2. Methods

This was a nonrandomized single-blind single-group clinical trial conducted as an extension of our previous trial [10].

### 2.1. Trial Population

We recruited all participants from Tartu University Hospital, Estonia. Our recruitment strategy included preliminary screening of medical histories of past 5 years of adult sick sinus syndrome patients with a permanent dual-chamber cardiac pacemaker for eligibility criteria, followed by inviting patients considered eligible to an outpatient visit to undergo final screening including a 12-lead electrocardiogram (ECG) and pacemaker interrogation [10].

### 2.2. Eligibility Criteria

The inclusion criteria were as follows: age 18–75 years; dual-chamber cardiac pacemaker implanted due to sick sinus syndrome at least 6 months earlier; and sinus rhythm confirmed by 12-lead ECG and pacemaker interrogation. The exclusion criteria were as follows: contraindication or intolerance to BBs, ivabradine, or their adjuvants; average seated office peripheral systolic BP ≥ 160 mmHg and/or diastolic BP ≥ 100 mmHg; atrial pacing < 40% or ventricular pacing > 25% since last pacemaker interrogation; unpaced QRS > 120 ms or QTc > 500 ms on 12-lead ECG; atrioventricular blockage at the atrial-paced-atrial-sensed (AAI) mode 90 bpm; resting HR > 60 bpm at the AAI mode 40 bpm; irregular HR on 12-lead ECG; frequent episodes of atrial high rate (automatic mode switching > 10%); implantable cardioverter defibrillator or cardiac resynchronization therapy pacemaker; treatment with digoxin or Vaughan-Williams class Ic or III antiarrhythmic drugs; history of acute coronary syndrome; stable angina pectoris; heart failure with reduced or preserved left ventricular ejection fraction; history of a cerebrovascular event; diabetes mellitus; thyroid gland disorders; chronic kidney disease with an estimated glomerular filtration rate of <30 ml/min/m^2^; symptomatic peripheral artery disease; clinically relevant heart valve disease; active cancer; acute or chronic inflammatory disease; severe chronic respiratory or liver disease; and pregnancy or breastfeeding.

### 2.3. Trial Design

**Figure 1:**
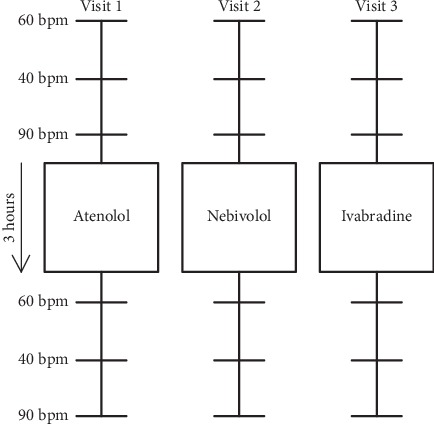
Trial design. At each visit, hemodynamic data were obtained before and at least three hours after drug ingestion at the middle, low, and high heart rate levels.

The trial design is presented in Figure 1. We performed all three trial visits in an outpatient setting after an overnight fast and abstinence from caffeine, tobacco, alcohol, and vigorous physical exercise by the trial participants. The participants were requested to discontinue their daily cardiovascular medication (except for antithrombotics) for 48 h before all visits, separated by a nonfixed interval of at least 1 week. At the first visit, we measured baseline sitting peripheral BP, obtained anthropometric data, blood samples, and baseline pacemaker parameters before commencing with trial interventions. Next, after at least 5 minutes of supine rest, we programmed the pacemaker to the atrial-paced-atrial-sensed (AAI) mode 60 bpm (middle HR level) and obtained hemodynamic data after 3 minutes of stabilization. After repeatedly collecting hemodynamic data at the AAI mode 40 bpm (low HR level) and then at the AAI mode 90 bpm (high HR level), we restored the baseline pacemaker settings and administered a 50 or 100 mg atenolol oral tablet to the participants. Hemodynamic measurements at the middle, at the low, and then at the high HR levels were replicated at least 3 hours after drug ingestion and sitting rest. We restored baseline pacemaker settings at the end of the visit. We repeated the above hemodynamic measurements at subsequent visits but administered a 5 mg nebivolol oral tablet to the participants at the second and a 5 or 7.5 mg ivabradine oral tablet at the third visit, i.e., all participants received all trial drugs in the same order. The participants were blinded to the trial drug sequence.

When a significant atrioventricular (AV) blockage appeared at the AAI mode 90 bpm, we reduced pacing rate at 5 bpm intervals up to 70 bpm. When frequent premature complexes appeared at the AAI modes 40, 60, or 90 bpm, we increased pacing rate at 5 bpm intervals up to 50, 70, or 95 bpm, respectively.

We performed all measurements under ECG monitoring. We made measurements at least in duplicate and averaged two closest results.

### 2.4. Hemodynamic Measurements

- Baseline BP, ECG monitoring, and pacemaker programming: we performed baseline BP measurement, ECG monitoring, and pacemaker programming using methods we described earlier [10].
- Pulse wave analysis and carotid-femoral pulse wave velocity (cf-PWV): we obtained peripheral and central hemodynamic indices noninvasively with a cuff-based oscillometric device (Sphygmocor XCEL software version 1.2, AtCor Medical, West Ride, Australia) as we described earlier [10]. All trial interventions and measurements were made by the same person (T. T.).

### 2.5. Laboratory Measurements

Blood samples collected at the first trial visit were analyzed using standard methods in a local clinical laboratory. We did not measure investigational drug concentrations in blood during the trial.

### 2.6. Statistical Analysis

The primary outcome measure was mean change in SBPA attained with atenolol at different HR levels. Other end points included mean changes in the following variables: SBPA with ivabradine and nebivolol, peripheral and central systolic and diastolic BP and pulse pressure (PP), mean arterial pressure (MAP), central augmentation pressure (AP), central augmentation index (AIx), pressure at the first and second systolic peaks of the central pressure waveform (P1 and P2, respectively), and cf-PWV. The baseline characteristics of the participants are presented as mean ± standard deviation or the median and interquartile ranges for continuous variables and as counts and percentages for categorical variables. Individual drug effects at different HR levels were assessed using one sample *t*-test. Differences in hemodynamic values obtained before drug administration, as well as differences in drug-induced changes in hemodynamic variables between three drugs and between three HR levels, were assessed with repeated measures ANOVA with the Bonferroni correction for multiple comparisons as appropriate. We did not perform power calculation to determine sample size. *P* < 0.05 was considered statistically significant. Statistical analysis was performed with the SAS software version 9.4.

The trial was registered in ClinicalTrials.gov at the beginning of the research process (NCT03245996). The trial protocol was approved by the Research Ethics Committee of the University of Tartu. We conducted the trial in accordance with the Declaration of Helsinki and with understanding and written consent of each participant.

## 3. Results

### 3.1. Subjects

In total, 142 of the 448 patients preliminarily screened were considered eligible to participate in the trial and were contacted by phone. 52 patients provided their informed consent and underwent final screening. Of them, 15 had screen failure, 2 withheld their consent, and 35 entered the trial visits [10]. During the trial, 4 participants withheld their consent, 5 were excluded due to significant AV blockage or frequent premature complexes not evident at screening and not responsive to pacing rate optimisation, and 1 due to violation of trial preparation instructions. 25 participants completed all three study visits and were included in data analysis. The trial visits were carried out from May 2015 to August 2016. Baseline characteristics of the participants are presented in Table 1.

### 3.2. Hemodynamic Data

Hemodynamic data before and after drug administration at the low, middle, and high HR levels are shown in Tables 2–4 and in Supplementary Tables 1–3, respectively.

The primary finding of this trial was that with atenolol, we observed a statistically significant decrease in SBPA at the low HR level, indicating a slightly smaller central systolic BP change compared to peripheral systolic BP change (mean change 0.74 ± 1.58 mmHg (95% confidence interval (CI), 0.09–1.40; *P* = 0.03)). At the middle and high HR levels, however, no significant change in SBPA occurred with atenolol (at the middle HR level, mean change −0.50 ± 1.53 mmHg (95% CI, −1.13–0.13; *P* = 0.11); at the high HR level, mean change 0.46 ± 1.78 mmHg (95% CI, −0.27–1.19; *P* = 0.21)). There was a statistically significant difference between SBPA change at the low and middle HR levels with atenolol, but we found no such difference between the low and high HR levels (inter-HR-level ANOVA, *P* < 0.01). The secondary outcomes were that unlike atenolol, nebivolol and ivabradine produced no significant change in SBPA at the low HR level (nebivolol, mean change −0.02 ± 2.40 mmHg (95% CI, −1.01–0.97; *P* = 0.99); ivabradine, mean change −0.48 ± 1.88 mmHg (95% CI, −1.26–0.30; *P* = 0.21)). Interdrug differences in SBPA change at the low HR level showed a trend towards significance in our small-volume trial (interdrug ANOVA, *P* = 0.06). Nebivolol produced no change in SBPA at the middle and high HR levels (at the middle HR level, mean change −0.20 ± 1.48 mmHg (95% CI, −0.81–0.41; *P* = 0.51); at the high HR level, mean change 0.12 ± 1.63 mmHg (95% CI, −0.55–0.79; *P* = 0.72)). Although there was a statistically significant increase in SBPA with ivabradine at the middle and no change at the high HR level, differences in SBPA change remained insignificant between different HR levels (at the middle HR level, mean change −1.02 ± 1.44 mmHg (95% CI, −1.61–(−0.43); *P* < 0.01); at the high HR level, mean change 0.62 ± 1.65 mmHg (95% CI, −1.30–0.06; *P* = 0.07); inter-HR-level ANOVA, *P* = 0.28)).

At the low HR level, all drugs significantly and similarly reduced AIx (interdrug ANOVA, *P*=0.93). Atenolol produced no significant change in AIx at the middle or high HR levels (inter-HR-level ANOVA, *P*=0.17).

Cf-PWV decreased significantly with atenolol at the low HR level only, in line with a significant decrease in MAP. At the middle and high HR levels, inferior MAP decrease compared to the low HR level probably explains no change in cf-PWV. There was no difference in cf-PWV change with atenolol between different HR levels (inter-HR-level ANOVA, *P*=0.56). The other drugs produced no change in cf-PWV at any HR level.

## 4. Discussion

We studied the effect of atenolol, nebivolol, and ivabradine on SBPA at different HR levels in a model of sick sinus syndrome patients with a permanent cardiac pacemaker in an acute setting. First, we showed a decrease in SBPA with atenolol at the low HR level, indicating an inferior central systolic BP change compared to peripheral systolic BP change. Second, elimination of atenolol-induced HR reduction with pacing resulted in no change in SBPA, i.e., no differential effect on central versus peripheral systolic BP at the middle and high HR levels. Third, pharmacological HR reduction with ivabradine or nebivolol at the low HR level did not change SBPA.

It has been debated whether the inferior ability of atenolol to reduce central compared to peripheral BP is related to its HR-dependent or HR-independent effects, or their combination [11–13]. HR reduction can lead to prolongation of ejection duration which increases the likelihood of a greater proportion of the reflected wave to appear in late systole and thus to augment central systolic BP. HR reduction-associated decrease in dP/dT (rate of pressure increase in early systole) can delay the time to the peak of the outgoing wave, increasing the chance of its coincidence with the reflected wave in late systole. HR reduction can improve diastolic filling of the left ventricle and increase ventricular stroke volume in a chronic setting, which, if ejected into a less compliant aorta (e.g., in elderly people), can also contribute to an increase in central systolic BP [14, 15]. Among HR-independent mechanisms, slight vasoconstriction, resulting from unopposed *α*-receptor stimulation or *ß*-2-receptor blockade, has been suggested to bring reflection sites more proximal to the aorta and thus to facilitate central systolic BP increase [9, 12].

BB- or ivabradine-treated sick sinus syndrome patients with a cardiac pacemaker serve as a model to mechanistically study the HR-dependent and HR-independent effects of BBs on hemodynamics. In our trial, the pacemaker's primary role at the low HR level was to ensure participant safety. Until the participant's HR remained over 40 bpm, the investigational drugs could exert their HR-lowering and other effects without any restriction by the pacemaker. The rationale of using the middle and high HR levels was to study drug effects in a situation where their pharmacological HR-lowering capacity had been nonpharmacologically eliminated by pacing, a relatively neutral method in hemodynamic terms, but the drugs' other effects, including HR-independent effects, on hemodynamics were preserved [16]. To our knowledge, elimination of BB- or ivabradine-induced HR reduction by pacing has only rarely been done earlier and for purposes different from ours [17–19]. Our trial thus adds a novel mechanistic insight into the topic of BBs, HR, and central hemodynamics.

Our finding that atenolol reduces central systolic BP less than peripheral systolic BP agrees with previous trials where atenolol, propranolol, and bisoprolol were used in an acute setting [11, 20–22]. This finding was recently confirmed in a meta-analysis summarising data of studies done in a chronic setting [7]. Furthermore, VBBs (mostly nebivolol) produced a similar reduction in central and peripheral systolic BP in this meta-analysis. We showed the same result with nebivolol at the low HR level in our acute study. We found no acute trials evaluating the effect of nebivolol on SBPA to compare with our study.

Ivabradine is a HR-lowering drug considered to be devoid of a direct effect on cardiac contractility/relaxation, AV conduction, or systemic vascular resistance [23, 24]. It thus enables to investigate the selective role of pharmacological HR reduction on hemodynamics. This has been done, to our knowledge, in a few small trials investigating mostly stable coronary artery disease patients and, excluding one study, in a more chronic setting compared to ours [25, 26]. Dillinger et al. reported that ivabradine resulted in no significant change in either peripheral or central systolic BP, similar to our study. They concluded that HR reduction *per se* does not impact central BP and hypothesized that inferior central BP reduction with NVBBs was associated with their HR-independent effects [9]. Rimoldi et al., on the contrary, suggested a negative relative impact of ivabradine-induced HR reduction on central versus peripheral BP [27]. The only acute study did not report data on peripheral systolic BP but found no central systolic BP change 3 hours after drug ingestion, similar to our study [26].

We found that ivabradine had no significantly different effect on central compared to peripheral systolic BP at the low HR level. We showed a similar phenomenon in our previous trial on the impact of nonpharmacological HR change on hemodynamics: an increase in HR from about 50 to 60 bpm by cardiac pacing produced no significant change in SBPA [10]. Hence, it could be mechanistically hypothesized that if atenolol had no HR-independent effects, e.g., on cardiac contractility or peripheral vasculature, it might perhaps not reduce central BP less than peripheral BP. Nebivolol, which impairs myocardial contractility less than atenolol, produced no change in SBPA, as we showed at the low HR level. However, it must be kept in mind that nebivolol's additional vasodilative effect could have contributed to central systolic BP decrease and that it reduced HR less than atenolol in our current trial [28]. Furthermore, atenolol resulted in a similar reduction in peripheral and central systolic BP at the middle and high HR levels, i.e., when pharmacological HR reduction was abolished by pacing, suggesting that HR-independent effects alone could also be insufficient to prevent central systolic BP to decrease as much as peripheral systolic BP. Only at the low HR level, when both HR-dependent and HR-independent effects were combined, did we observe an inferior central vs peripheral systolic BP decrease with atenolol.

This trial has several limitations. It was a single-center small-volume trial performed in an acute setting, the results and conclusions of which need to be interpreted with caution. We did not measure investigational drug blood concentrations. Also, at the low HR level, many participants were paced after drug administration, indicating that the drugs could not exert their full HR-lowering effect. However, this was due to safety reasons. Additionally, absence of randomization resulted in higher BP and MAP at the first visit, which could have influenced hemodynamic outcomes attained with atenolol. Also, at the high HR level, we could not maintain the exact predrug HR value after BB ingestion, since the pacing rate had to be reduced in many participants due to BB-induced AV blockage. Furthermore, our results obtained with atenolol and nebivolol cannot be extrapolated to populations different from ours and to all NVBBs or to all VBBs, respectively, since BBs are a highly heterogeneous class of drugs. Interpretation of our results may also be hampered by the notion that ivabradine can have pleiotropic effects on hemodynamics beyond HR reduction [29, 30].

## 5. Conclusions

One of the reasons for weaker cardiovascular protection with atenolol compared to other drug classes in uncomplicated hypertension can be its inferior ability to reduce central versus peripheral BP. The findings of our trial with a mechanistic approach to the topic suggest that it is the combination of atenolol's HR-dependent and HR-independent effects that this phenomenon could be explained by rather than either effect alone.

## Figures and Tables

**Table 1:** Baseline characteristics.

Characteristic	Values
Demographic characteristics	
Age, years	65.5 ± 9.8
Male sex, *n* (%)	12 (48)
Height, cm	165.8 ± 8.2
Body mass index (kg/m^2^)	29.5 ± 4.1

Disease characteristics, *n* (%)	
Hypertension	17 (68)
Coronary artery disease	4 (16)
Dyslipidemia	23 (92)
Atrial fibrillation	10 (40)
Chronic kidney disease (eGFR 30–60 ml/m^2^/1.73 m^2^)	3 (12)

Pacemaker characteristics	
Atrial pacing (%)	85.0 (64.0–93.0)
Ventricular pacing < 1%, *n* (%)	14 (56)

Cardiovascular medication, *n* (%)	
Treatment-naïve	6 (28)
ACE inhibitors/ARBs	8 (32)
Calcium channel blockers	4 (16)
Diuretics	5 (20)
*ß*-Blockers	12 (48)
Statins	4 (16)
Antiplatelets	8 (32)
Anticoagulants	2 (8)

Blood analyses	
Glucose (mmol/L)	5.4 ± 0.7
Total cholesterol (mmol/L)	5.8 ± 1.2
LDL cholesterol (mmol/L)	3.9 ± 1.2
HDL cholesterol (mmol/L)	1.6 ± 0.5
Triglycerides (mmol/L)	1.5 ± 0.5
eGFR (mL/min/1.73 m^2^)	78.5 ± 16.2
NT-proBNP (pg/mL)	148.0 (85.0–186.0)
hsCRP (mg/L)	1.6 (1.0–2.6)

Hemodynamic characteristics	
Peripheral systolic BP (mmHg)	127.8 ± 14.9
Peripheral diastolic BP (mmHg)	80.8 ± 10.0
Heart rate (bpm)	64.4 ± 3.9

Others	
Current smoking, *n* (%)	5 (20)

Values are presented as mean ± SD, median (interquartile range), or count (%). eGFR indicates estimated glomerular filtration rate; ACE, angiotensin-converting enzyme; ARB, angiotensin receptor blocker; LDL, low-density lipoprotein; HDL, high-density lipoprotein; NT-proBNP, *N*-terminal pro-brain natriuretic peptide; hsCRP, high-sensitivity C-reactive protein; and BP, blood pressure.

**Table 2:** Effect of *ß*-blockers and ivabradine on hemodynamic parameters at the low heart rate level (AAI 40 bpm).

Parameter	Atenolol	Nebivolol	Ivabradine	*P* value for baselines	*P* value for difference
Heart rate, bpm					
Baseline	48.64 ± 4.79	48.60 ± 5.25	49.58 ± 5.41	0.45	
End	43.66 ± 3.53	47.02 ± 5.32	46.46 ± 4.95		
Difference from baseline to end (*P* value; 95% CI)	4.98 ± 3.98 (<0.01; 3.34–6.62)	1.58 ± 2.59 (<0.01; 0.51–2.65)	3.12 ± 4.37 (<0.01; 1.32–4.92)		<0.01 (ate similar to ivb, neb similar to ivb)

Peripheral systolic blood pressure, mmHg					
Baseline	129.64 ± 15.09	126.42 ± 13.86	126.46 ± 13.24	0.11	
End	119.74 ± 14.95	121.94 ± 12.24	123.58 ± 11.42		
Difference from baseline to end (*P* value; 95% CI)	9.90 ± 11.99 (<0.01; 4.95–14.85)	4.48 ± 7.91 (<0.01; 1.22–7.74)	2.88 ± 8.92 (0.12; −0.80–6.56)		0.02 (ate similar to neb, neb similar to ivb)

Central systolic blood pressure, mmHg					
Baseline	120.08 ± 14.87	117.38 ± 13.11	117.66 ± 12.62	0.17	
End	110.92 ± 14.09	112.88 ± 12.22	114.30 ± 11.80		
Difference from baseline to end (*P* value; 95% CI)	9.16 ± 11.81 (<0.01; 4.28–14.04)	4.50 ± 7.62 (<0.01; 1.36–7.64)	3.36 ± 8.65 (0.06; −0.21–6.93)		0.06

Systolic blood pressure amplification, mmHg					
Baseline	9.56 ± 2.70	9.04 ± 3.34	8.8 ± 2.94	0.28	
End	8.82 ± 2.76	9.06 ± 2.72	9.28 ± 2.36		
Difference from baseline to end (*P* value; 95% CI)	0.74 ± 1.58 (0.03; 0.09–1.40)	−0.02 ± 2.40 (0.99; −1.01–0.97)	−0.48 ± 1.88 (0.21; −1.26–0.30)		0.06

Values are presented as mean ± SD. Ate indicates atenolol; ivb, ivabradine; and neb, nebivolol.

**Table 3:** Effect of *ß*-blockers and ivabradine on hemodynamic parameters at the middle heart rate level (AAI 60 bpm).

Parameter	Atenolol	Nebivolol	Ivabradine	*P* value for baselines	*P* value for difference
Heart rate (bpm)					
Baseline	60.38 ± 1.15	60.12 ± 0.60	60.16 ± 0.62	0.26	
End	60.28 ± 1.06	60.12 ± 0.60	60.08 ± 0.43		
Difference from baseline to end (*P* value; 95% CI)	0.10 ± 0.29 (0.1; −0.02–0.22)	0.00 ± 0.00 (–)	0.08 ± 0.31 (0.21; −0.05–0.21)		0.30

Peripheral systolic blood pressure (mmHg)					
Baseline	134.54 ± 13.64	130.62 ± 13.87	130.4 ± 15.08	0.09	
End	130.4 ± 14.35	128.42 ± 12.65	129.44 ± 10.05		
Difference from baseline to end (*P* value; 95% CI)	4.14 ± 10.17 (0.05; 0.06–8.34)	2.20 ± 9.38 (0.25; −1.67–6.07)	0.96 ± 10.04 (0.64; −3.19–5.11)		0.47

Central systolic blood pressure (mmHg)					
Baseline	124.86 ± 13.18	121.38 ± 13.48	121.24 ± 13.93	0.10	
End	120.22 ± 13.08	118.98 ± 11.79	119.26 ± 9.9		
Difference from baseline to end (*P* value; 95% CI)	4.64 ± 9.95 (0.03; 0.53–8.75)	2.40 ± 9.31 (0.21; −1.44–6.24)	1.98 ± 9.65 (0.32; −2.00–5.96)		0.50

Systolic blood pressure amplification (mmHg)					
Baseline	9.68 ± 2.68	9.24 ± 3.14	9.16 ± 2.97	0.41	
End	10.18 ± 2.51	9.44 ± 3.04	10.18 ± 2.52		
Difference from baseline to end (*P* value; 95% CI)	−0.50 ± 1.53 (0.11; −1.13–0.13)	−0.20 ± 1.48 (0.51; −0.81–0.41)	−1.02 ± 1.44 (<0.01; −1.61–(−0.43))		0.14

Values are presented as mean ± SD.

**Table 4:** Effect of *ß*-blockers and ivabradine on hemodynamic parameters at the high heart rate level (AAI 90 bpm).

Parameter	Atenolol	Nebivolol	Ivabradine	*P* value for baselines	*P* value for difference
Heart rate (bpm)					
Baseline	87.00 ± 4.48	86.88 ± 5.59	87.04 ± 5.00	0.97	
End	84.96 ± 6.09	85.12 ± 7.05	85.58 ± 6.38		
Difference from baseline to end (*P* value; 95% CI)	2.04 ± 3.77 (0.01; 0.48–3.60)	1.76 ± 3.55 (0.02; 0.29–3.23)	1.46 ± 3.82 (0.07; −0.12–3.04)		0.83

Peripheral systolic blood pressure (mmHg)					
Baseline	136.96 ± 11.60	131.52 ± 13.05	131.62 ± 14.44	0.01; neb similar to ivb	
End	131.02 ± 11.70	129.26 ± 11.66	131.38 ± 11.07		
Difference from baseline to end (*P* value; 95% CI)	5.94 ± 10.02 (<0.01; 1.80–10.08)	2.26 ± 6.98 (0.12; −0.62–5.14)	0.24 ± 9.46 (0.90; −3.67–4.15)		0.047; ate similar to neb, neb similar to ivb

Central systolic blood pressure (mmHg)					
Baseline	124.84 ± 10.95	119.76 ± 11.70	120.3 ± 13.30	0.01; neb similar to ivb	
End	119.36 ± 10.91	117.62 ± 11.00	119.44 ± 11.15		
Difference from baseline to end (*P* value; 95% CI)	5.48 ± 9.97 (0.01; 1.36–9.60)	2.14 ± 6.41 (0.11; −0.50–4.78)	0.86 ± 8.79 (0.63; −2.77–4.49)		0.09

Systolic blood pressure amplification (mmHg)					
Baseline	12.12 ± 2.45	11.76 ± 2.77	11.32 ± 2.93	0.09	
End	11.66 ± 2.11	11.64 ± 2.07	11.94 ± 3.13		
Difference from baseline to end (*P* value; 95% CI)	0.46 ± 1.78 (0.21; −0.27–1.19)	0.12 ± 1.63 (0.72; −0.55–0.79)	−0.62 ± 1.65 (0.07; −1.30–0.06)		0.09

Values are presented as mean ± SD. Neb indicates nebivolol; ivb, ivabradine; and ate, atenolol.

## Data Availability

The data used to support the findings of this study are available from the corresponding author upon request.

## Supplementary Materials

Supplementary Materials This file contains three supplementary tables. Supplementary Table 1: effect of *ß*-blockers and ivabradine on hemodynamic parameters at the low heart rate level (AAI 40 bpm). Supplementary Table 2: effect of *ß*-blockers and ivabradine on hemodynamic parameters at the middle heart rate level (AAI 60 bpm). Supplementary Table 3: effect of *ß*-blockers and ivabradine on hemodynamic parameters at the high heart rate level (AAI 90 bpm).

## References

[1] Whelton P. K., Carey R. M., Aronow W. S. (2018). 2017 ACC/AHA/AAPA/ABC/ACPM/AGS/APhA/ASH/ASPC/NMA/PCNA guideline for the prevention, detection, evaluation, and management of high blood pressure in adults: a report of the American College of Cardiology/American Heart Association task force on clinical practice guidelines. *Hypertension*.

[2] National Institute for Health and Clinical Excellence Hypertension: clinical management of primary hypertension in adults (update). http://guidance.nice.org.uk/CG127.

[3] Williams B., Mancia G., Spiering W. (2018). 2018 ESC/ESH guidelines for the management of arterial hypertension. *Journal of Hypertension*.

[4] Williams B., Lacy P. S., Thom S. M. (2006). Differential impact of blood pressure–lowering drugs on central aortic pressure and clinical outcomes. *Circulation*.

[5] Ding F. H., Li Y., Li L. H. (2013). Impact of heart rate on central hemodynamics and stroke: a meta-analysis of *β*-blocker trials. *American Journal of Hypertension*.

[6] Dalal J., Dasbiswas A., Sathyamurthy I. (2019). Heart rate in hypertension: review and expert opinion. *International Journal of Hypertension*.

[7] Pucci G., Ranalli M. G., Battista F. (2016). Effects of *β*-blockers with and without vasodilating properties on central blood pressure. *Hypertension*.

[8] Kampus P., Serg M., Kals J. (2011). Differential effects of nebivolol and metoprolol on central aortic pressure and left ventricular wall thickness. *Hypertension*.

[9] Dillinger J. G., Maher V., Vitale C. (2015). Impact of ivabradine on central aortic blood pressure and myocardial perfusion in patients with stable coronary artery disease. *Hypertension*.

[10] Teeäär T., Serg M., Paapstel K. (2018). Heart rate reduction decreases central blood pressure in sick sinus syndrome patients with a permanent cardiac pacemaker. *Journal of Human Hypertension*.

[11] Cockburn J. A., Brett S. E., Guilcher A. (2010). Differential effects of betaadrenoreceptor antagonists on central and peripheral blood pressure at rest and during exercise. *British Journal of Clinical Pharmacology*.

[12] Goupil R., Dupuis D., Troyanov S. (2016). Heart rate dependent and independent effects of beta-blockers on central hemodynamic parameters. *Journal of Hypertension*.

[13] Messerli F. H., Rimoldi S. F., Bangalore S. (2016). When an increase in central systolic pressure overrides the benefits of heart rate lowering. *Journal of the Americal College of Cardiology*.

[14] Williams B., Lacy P. S. (2009). Impact of heart rate on central aortic pressures and hemodynamics:. *Journal of the Americal College of Cardiology*.

[15] Lund-Johansen P. (1976). Haemodynamic long-term effects of a new beta-adrenoceptor blocking drug, atenolol (ICI 66082), in essential hypertension. *British Journal of Clinical Pharmacology*.

[16] Tan I., Kiat H., Barin E. (2016). Effects of pacing modality on non-invasive assessment of heart rate dependency of indices of large artery function. *Journal of Applied Physiology*.

[17] Liang Y. L., Gatzka C. D., Du X. J. (1999). Effects of heart rate on arterial compliance in men. *Clinical and Experimental Pharmacology and Physiology*.

[18] Thackray S. D. R., Ghosh J. M., Wright G. A. (2006). The effect of altering heart rate on ventricular function in patients with heart failure treated with beta-blockers. *American Heart Journal*.

[19] Heusch G., Skyschally A., Gres P. (2008). Improvement of regional myocardial blood flow and function and reduction of infarct size with ivabradine: protection beyond heart rate reduction. *European Heart Journal*.

[20] Hirata K., Vlachopoulos C., Adji A. (2005). Benefits from angiotensin-converting enzyme inhibitor “beyond blood pressure lowering”: beyond blood pressure or beyond the brachial artery?. *Journal of Hypertension*.

[21] Casey D. P., Curry T. B., Joyner M. J. (2012). Acute *β*-adrenergic blockade increases aortic wave reflection in young men and women. *Hypertension*.

[22] Adji A., Hirata K., Hoegler S. (2007). Noninvasive pulse waveform analysis in clinical trials: similarity of two methods for calculating aortic systolic pressure. *American Journal of Hypertension*.

[23] Koruth J. S., Lala A., Pinney S. (2017). The clinical use of ivabradine. *Journal of the Americal College of Cardiology*.

[24] Rognoni A., Bertolazzi M., Macciò S. (2009). Ivabradine: cardiovascular effects. *Recent Patents of Cardiovascular Drug Discovery*.

[25] Lopatin Y. M., Vitale C. (2016). Effect of ivabradine on central aortic blood pressure in patients with stable coronary artery disease: what do we know?. *International Journal of Cardiology*.

[26] Lopatin Y. M., Uskova V. A. (2015). The acute and chronic effects of ivabradine on the parameters of central aortic pressure in patients with stable coronary artery disease. *European Heart Journal*.

[27] Rimoldi S. F., Messerli F. H., Cerny D. (2016). Selective heart rate reduction with ivabradine increases central blood pressure in stable coronary artery disease. *Hypertension*.

[28] Mangrella M., Rossi F., Fici F. (1998). Pharmacology of nebivolol. *Pharmacological Research*.

[29] Heusch G., Kleinbongard P. (2016). Ivabradine: cardioprotection by and beyond heart rate reduction. *Drugs*.

[30] Fischer-Rasokat U., Honold J., Lochmann D. (2014). Ivabradine therapy to unmask heart rate-independent effects of *β*-blockers on pulse wave reflections. *Clinical Research in Cardiology*.

