# Supplementary material for: Atenolol's Inferior Ability to Reduce Central vs Peripheral Blood Pressure Can Be Explained by the Combination of Its Heart Rate-Dependent and Heart Rate-Independent Effects
Source: Int J Hypertens. 2020 Apr 26;2020:4259187. doi: 10.1155/2020/4259187 (PMC7201670; doi:10.1155/2020/4259187)
Supplement: Supplementary Materials — This file contains three supplementary tables. Supplementary Table 1: effect of ß-blockers and ivabradine on hemodynamic parameters at the low heart rate level (AAI 40 bpm). Supplementary Table 2: effect of ß-blockers and ivabradine on hemodynamic parameters at the middle heart rate level (AAI 60 bpm). Supplementary Table 3: effect of ß-blockers and ivabradine on hemodynamic parameters at the high heart rate level (AAI 90 bpm). [file 4259187.f1.doc]

**SUPPLEMENTARY MATERIALS**

This file contains three supplementary tables.

Supplementary Table 1. Effect of ß-blockers and ivabradine on hemodynamic parameters at the low heart rate level (AAI 40 bpm)

| **Parameter** | **Atenolol** | **Nebivolol** | **Ivabradine** | **P-Value for baselines** | **P-Value**  **for difference** |
| --- | --- | --- | --- | --- | --- |
| **Peripheral diastolic blood pressure, mmHg** | | | | | |
| -Baseline | 75.88 ± 9.2 | 72.86 ± 7.43 | 72.98 ± 7.63 | 0.02  Similar:  -neb/ivb |  |
| -End | 70.06 ± 9.24 | 71.24 ± 7.2 | 70.62 ± 7.54 |  |  |
| -Difference from baseline to End (P-value; 95% CI) | 5.82 ± 8.96  (<0.01; 2.12 – 9.52) | 1.62 ± 4.56  (0.09; -0.26 –3.50) | 2.36 ± 6.89  (0.10; -0.48 –5.20) |  | 0.06 |
| **Peripheral pulse pressure, mmHg** | | | | | |
| -Baseline | 53.76 ± 9.59 | 53.3 ± 9.85 | 53.48 ± 9.47 | 0.94 |  |
| -End | 49.68 ± 8.48 | 50.7 ± 8.15 | 52.96 ± 7.98 |  |  |
| -Difference from baseline to end (P-value; 95% CI) | 4.08 ± 6.64  (<0.01; 1.34 – 6.82) | 2.60 ± 4.79  (0.01; 0.62 – 4.58) | 0.52 ± 5.53  (0.64; -1.76 –2.80) |  | 0.09 |
| **Central diastolic blood pressure, mmHg** | | | | | |
| -Baseline | 76.48 ± 9.38 | 73.32 ± 7.52 | 73.54 ± 7.68 | 0.01  Similar:  -neb/ivb |  |
| -End | 70.5 ± 9.35 | 71.7 ± 7.33 | 71.08 ± 7.56 |  |  |
| -Difference from baseline to end (P-value; 95% CI) | 5.98 ± 9.16  (<0.01; 2.20 – 9.76) | 1.62 ± 4.57  (0.09; -0.27 – 3.51) | 2.46 ± 6.87  (0.09; -0.37 – 5.29) |  | 0.05 |
| **Mean arterial pressure, mmHg** | | | | | |
| -Baseline | 91.02 ± 10.81 | 88 ± 8.88 | 88.24 ± 8.81 | 0.03  Similar:  -ate/ivb  -neb/ivb |  |
| -End | 83.97 ± 10.6 | 85.42 ± 8.6 | 85.49 ± 8.45 |  |  |
| -Difference from baseline to end (P-value; 95% CI) | 7.05 ± 9.78  (<0.01; 3.02 – 11.09) | 2.58 ± 5.40  (0.03; 0.35 – 4.81) | 2.75 ± 7.23  (0.07; -0.23 – 5.74) |  | 0.05 |
| **Central pulse pressure, mmHg** | | | | | |
| -Baseline | 43.64 ± 8.42 | 44.06 ± 8.53 | 44.06 ± 8.16 | 0.91 |  |
| -End | 40.44 ± 7.34 | 41.16 ± 7.25 | 43.16 ± 7.63 |  |  |
| -Difference from baseline to end (P-value; 95% CI) | 3.2 ± 5.59  (<0.01; 0.89 – 5.51) | 2.90 ± 4.31  (<0.01; 1.12 – 4.68) | 0.9 ± 4.35  (0.31; -0.90 – 2.70) |  | 0.20 |
| **Central augmentation index, %** | | | | | |
| -Baseline | 24.94 ± 9.26 | 26.44 ± 10.86 | 27.56 ± 10.51 | 0.21 |  |
| -End | 21.36 ± 9.4 | 22.44 ± 10.66 | 23.52 ± 9.38 |  |  |
| -Difference from baseline to end (P-value; 95% CI) | 3.58 ± 4.89  (<0.01; 1.56 – 5.60) | 4.00 ± 5.51  (<0.01; 1.73 – 6.27) | 4.04 ± 5.75  (<0.01; 1.67 – 6.4) |  | 0.93 |
| **Central augmentation pressure, mmHg** | | | | | |
| -Baseline | 11.36 ± 5.43 | 12 ± 5.61 | 12.44 ± 5.34 | 0.41 |  |
| -End | 8.92 ± 4.24 | 9.58 ± 5.11 | 10.6 ± 5.25 |  |  |
| -Difference from baseline to end (P-value; 95% CI) | 2.44 ± 3.25  (<0.01; 1.10 – 3.78) | 2.42 ± 3.09  (<0.01; 1.14 – 3.70) | 1.84 ± 3.02  (<0.01; 0.59 – 3.09) |  | 0.7 |
| **P1, mmHg** | | | | | |
| -Baseline | 108.72 ± 10.92 | 105.38 ± 10.1 | 105.2 ± 10.39 | 0.03  Similar:  -ate/neb  -neb/ivb |  |
| -End | 101.94 ± 12.57 | 103.26 ± 9.7 | 103.7 ± 8.68 |  |  |
| -Difference from baseline to end (P-value; 95% CI) | 6.78 ± 9.83  (<0.01; 2.72 – 10.84) | 2.12 ± 5.95  (0.09; -0.33 – 4.57) | 1.5 ± 6.93  (0.29; -1.36 – 4.36) |  | 0.03  Similar:  -ate/neb  -neb/ivb |
| **P2, mmHg** | | | | | |
| -Baseline | 120.08 ± 14.87 | 117.38 ± 13.11 | 117.64 ± 12.67 | 0.17 |  |
| -End | 110.86 ± 14.2 | 112.84 ± 12.31 | 114.3 ± 11.8 |  |  |
| -Difference from baseline to end (P-value; 95% CI) | 9.22 ± 11.8  (<0.01; 4.35 – 14.09) | 4.54 ± 7.60  (<0.01; 1.40 – 7.68) | 3.34 ± 8.67  (0.07; -0.24 – 6.92) |  | 0.05 |
| **Cf-PWV, m/s** | | | | | |
| -Baseline | 8.49 ± 2.67 | 8.16 ± 2.11 | 8.27 ± 2.13 | 0.10 |  |
| -End | 7.92 ± 2.13 | 8.15 ± 2.16 | 8.09 ± 2.09 |  |  |
| -Difference from baseline to end (P-value; 95% CI) | 0.57 ± 1.00  (<0.01; 0.15 – 0.98) | 0.01 ± 0.56  (0.93; -0.22 – 0.24) | 0.18 ± 0.56  (0.12; -0.05 – 0.42) |  | <0.01  Similar:  -ate/ivb  -neb/ivb |

Values are presented as mean ± SD. P1 indicates pressure at the first systolic peak of the central waveform; P2, pressure at the second systolic peak of the central waveform; cf-PWV, carotid-femoral pulse wave velocity; neb, nebivolol; ivb, ivabradine; and ate, atenolol.

Supplementary Table 2. Effect of ß-blockers and ivabradine on hemodynamic parameters at the middle heart rate level (AAI 60 bpm)

| **Parameter** | **Atenolol** | **Nebivolol** | **Ivabradine** | **P-Value for baselines** | **P-Value**  **For difference** |
| --- | --- | --- | --- | --- | --- |
| **Peripheral diastolic blood pressure, mmHg** | | | | | |
| -Baseline | 81.52 ± 7.03 | 78.94 ± 8.47 | 78.62 ± 8.8 | 0.04  Similar:  -neb/ivb |  |
| -End | 78.86 ± 9.42 | 78.78 ± 7.91 | 78.6 ± 8.2 |  |  |
| -Difference from baseline to end (P-value; 95% CI) | 2.66 ± 6.07  (0.04; 0.15 – 5.17) | 0.16 ± 6.04  (0.90; -2.34 – 2.66) | 0.02 ± 7.98  (0.99; -3.27 – 3.31) |  | 0.25 |
| **Peripheral pulse pressure, mmHg** | | | | | |
| -Baseline | 53.02 ± 9.33 | 51.68 ± 8.2 | 51.74 ± 10.12 | 0.59 |  |
| -End | 51.54 ± 8.15 | 49.66 ± 9.15 | 50.86 ± 7.86 |  |  |
| -Difference from baseline to end (P-value; 95% CI) | 1.48 ± 5.85  (0.22; -0.93 – 3.89) | 2.02 ± 5.68  (0.09; -0.32 – 4.36) | 0.88 ± 5.56  (0.44; -1.41 – 3.17) |  | 0.76 |
| **Central diastolic blood pressure, mmHg** | | | | | |
| -Baseline | 82.46 ± 7.1 | 79.88 ± 8.48 | 79.56 ± 8.95 | 0.04  Similar:  -neb/ivb |  |
| -End | 79.86 ± 9.38 | 79.7 ± 7.97 | 79.42 ± 8.21 |  |  |
| -Difference from baseline to end (P-value; 95% CI) | 2.60 ± 6.06  (0.04; 0.10 – 5.10) | 0.18 ± 5.91  (0.88; -2.26 – 2.62) | 0.14 ± 7.94  (0.93; -3.14 – 3.42) |  | 0.28 |
| **Mean arterial pressure, mmHg** | | | | | |
| -Baseline | 96.59 ± 8.7 | 93.71 ± 9.87 | 93.45 ± 10.1 | 0.04  Similar:  -neb/ivb |  |
| -End | 93.3 ± 10.26 | 92.79 ± 8.76 | 92.7 ± 8.24 |  |  |
| -Difference from baseline to end (P-value; 95% CI) | 3.29 ± 7.11  (0.03; 0.35 – 6.22) | 0.92 ± 6.77  (0.50; -1.88 – 3.72) | 0.76 ± 8.26  (0.65; -2.65 – 4.16) |  | 0.34 |
| **Central pulse pressure, mmHg** | | | | | |
| -Baseline | 42.44 ± 8.43 | 41.52 ± 7.08 | 41.72 ± 8.54 | 0.72 |  |
| -End | 40.34 ± 6.79 | 39.24 ± 7.36 | 39.86 ± 6.61 |  |  |
| -Difference from baseline to end (P-value; 95% CI) | 2.10 ± 5.66  (0.08; -0.24 – 4.44) | 2.28 ± 5.33  (0.04; 0.08 – 4.48) | 1.86 ± 4.76  (0.06; -0.10 – 3.82) |  | 0.95 |
| **Central augmentation index, %** | | | | | |
| -Baseline | 28.1 ± 8.53 | 29.46 ± 11.7 | 29.2 ± 10.26 | 0.51 |  |
| -End | 26.12 ± 9.5 | 26.36 ± 9.56 | 24.64 ± 8.92 |  |  |
| -Difference from baseline to end (P-value; 95% CI) | 1.98 ± 6.44  (0.14; -0.68 – 4.64) | 3.10 ± 6.35  (0.02; 0.48 – 5.72) | 4.56 ± 5.07  (<0.01; 2.47 – 6.65) |  | 0.23 |
| **Central augmentation pressure, mmHg** | | | | | |
| -Baseline | 12.32 ± 5.56 | 12.54 ± 5.85 | 12.5 ± 5.75 | 0.94 |  |
| -End | 10.52 ± 4.02 | 10.38 ± 4.26 | 10.02 ± 4.28 |  |  |
| -Difference from baseline to end (P-value; 95% CI) | 1.8 ± 3.85  (0.03; 0.21 – 3.39) | 2.16 ± 3.93  (0.01; 0.54 – 3.78) | 2.48 ± 3.06  (<0.01; 1.22 – 3.74) |  | 0.72 |
| **P1, mmHg** | | | | | |
| -Baseline | 112.54 ± 9.79 | 108.84 ± 10.84 | 108.74 ± 12.24 | 0.04  Similar:  -neb/ivb |  |
| -End | 109.7 ± 12.14 | 108.6 ± 9.95 | 109.24 ± 8.42 |  |  |
| -Difference from baseline to end (P-value; 95% CI) | 2.84 ± 8.11  (0.09; -0.51 – 6.19) | 0.24 ± 7.08  (0.87; -2.68 – 3.16) | -0.5 ± 8.36  (0.77; -3.95 – 2.95) |  | 0.26 |
| **P2, mmHg** | | | | | |
| -Baseline | 124.86 ± 13.18 | 121.38 ± 13.48 | 121.24 ± 13.93 | 0.10 |  |
| -End | 120.22 ± 13.08 | 118.98 ± 11.79 | 119.26 ± 9.9 |  |  |
| -Difference from baseline to end (P-value; 95% CI) | 4.64 ± 9.95  (0.03; 0.53 – 8.75) | 2.4 ± 9.31  (0.21; -1.44 – 6.24) | 1.98 ± 9.65  (0.32; -2.00 – 5.69) |  | 0.50 |
| **Cf-PWV, m/s** | | | | | |
| -Baseline | 9.06 ± 2.39 | 8.8 ± 2.36 | 8.82 ± 2.18 | 0.18 |  |
| -End | 8.69 ± 2.36 | 8.67 ± 2.2 | 8.68 ± 1.97 |  |  |
| -Difference from baseline to end (P-value; 95% CI) | 0.36 ± 1.15  (0.13; -0.11 – 0.84) | 0.13 ± 0.8  (0.42; -0.20 – 0.46) | 0.14 ± 0.83  (0.40; -0.20 – 0.48) |  | 0.56 |

Values are presented as mean ± SD. P1 indicates pressure at the first systolic peak of the central waveform; P2, pressure at the second systolic peak of the central waveform; cf-PWV, carotid-femoral pulse wave velocity; neb, nebivolol; and ivb, ivabradine.

Supplementary Table 3. Effect of ß-blockers and ivabradine on hemodynamic parameters at the high heart rate level (AAI 90 bpm)

| **Parameter** | **Atenolol** | **Nebivolol** | **Ivabradine** | **P-Value for baselines** | **P-Value**  **for difference** |
| --- | --- | --- | --- | --- | --- |
| **Peripheral diastolic blood pressure, mmHg** | | | | | |
| -Baseline | 90.48 ± 7.82 | 86.66 ± 9.12 | 86.64 ± 8.29 | 0.003  Similar:  -neb/ivb |  |
| -End | 86.7 ± 9.83 | 85.38 ± 9.03 | 86.3 ± 9.76 |  |  |
| - Difference from baseline to end (P-value; 95% CI) | 3.78 ± 8.93  (0.04; 0.10 – 7.46) | 1.28 ± 6.07  (0.30; -1.23 – 3.79) | 0.34 ± 6.11  (0.78; -2.18 – 2.86) |  | 0.16 |
| **Peripheral pulse pressure, mmHg** | | | | | |
| -Baseline | 46.48 ± 7.42 | 44.86 ± 7.44 | 44.98 ± 8.2 | 0.43 |  |
| -End | 44.32 ± 6.86 | 43.88 ± 5.82 | 45.06 ± 6.5 |  |  |
| - Difference from baseline to end (P-value; 95% CI) | 2.16 ± 4.35  (0.02; 0.36 – 3.96) | 0.98 ± 5.73  (0.40; -1.39 – 3.35) | -0.08 ± 5.93  (0.95; -2.53 – 2.37) |  | 0.35 |
| **Central diastolic blood pressure, mmHg** | | | | | |
| -Baseline | 91.38 ± 8 | 87.68 ± 9.18 | 87.62 ± 8.41 | 0.005  Similar:  -neb/ivb |  |
| -End | 87.56 ± 9.69 | 86.34 ± 8.79 | 87.2 ± 10.12 |  |  |
| - Difference from baseline to end (P-value; 95% CI) | 3.82 ± 8.93  (0.04; 0.14 – 7.50) | 1.34 ± 5.92  (0.27; -1.10 – 3.78) | 0.42 ± 6.10  (0.73; -2.10 – 2.94) |  | 0.17 |
| **Mean arterial pressure, mmHg** | | | | | |
| -Baseline | 102.54 ± 8.6 | 98.36 ± 9.73 | 98.52 ± 9.78 | 0.004  Similar:  -neb/ivb |  |
| -End | 98.16 ± 9.76 | 96.77 ± 9.34 | 97.94 ± 10.15 |  |  |
| - Difference from baseline to end (P-value; 95% CI) | 4.38 ± 9.15  (0.02; 0.60 – 8.16) | 1.59 ± 5.69  (0.17; -0.76 – 3.94) | 0.58 ± 6.73  (0.67; -2.20 – 3.36) |  | 0.11 |
| **Central pulse pressure, mmHg** | | | | | |
| -Baseline | 33.46 ± 6.19 | 32.04 ± 5.64 | 32.8 ± 6.98 | 0.47 |  |
| -End | 31.9 ± 5.57 | 31.36 ± 4.54 | 34.96 ± 14.25 |  |  |
| - Difference from baseline to end (P-value; 95% CI) | 1.56 ± 3.36  (0.03; 0.17 – 2.95) | 0.68 ± 4.54  (0.46; -1.20 – 2.56) | -2.16 ± 12.83  (0.41; -7.46 – 3.14) |  | 0.27 |
| **Central augmentation index, %** | | | | | |
| -Baseline | 19.48 ± 7.76 | 18.28 ± 8.63 | 19.76 ± 11.06 | 0.42 |  |
| -End | 18.46 ± 7.87 | 17.52 ± 8.38 | 17.24 ± 10.82 |  |  |
| - Difference from baseline to end (P-value; 95% CI) | 1.02 ± 5.42  (0.36; -1.22 – 3.26) | 0.76 ± 4.56  (0.41; -1.12 – 2.64) | 2.52 ± 6.93  (0.08; 2.52 – 6.93) |  | 0.53 |
| **Central augmentation pressure, mmHg** | | | | | |
| -Baseline | 6.96 ± 3.47 | 6.18 ± 3.23 | 6.92 ± 4.33 | 0.38 |  |
| -End | 6.06 ± 3.02 | 5.72 ± 3.09 | 5.92 ± 4.31 |  |  |
| - Difference from baseline to end (P-value; 95% CI) | 0.9 ± 2.71  (0.11; -0.22 – 2.02) | 0.46 ± 2.14  (0.29; -0.42 – 1.34) | 1.00 ± 2.85  (0.09; -0.18 – 2.18) |  | 0.74 |
| **P1, mmHg** | | | | | |
| -Baseline | 117.88 ± 9.3 | 113.58 ± 10.71 | 113.2 ± 11.09 | 0.007  Similar:  -neb/ivb | 2 |
| -End | 113.3 ± 10.61 | 111.86 ± 9.99 | 113.46 ± 10.43 |  |  |
| - Difference from baseline to end (P-value; 95% CI) | 4.58 ± 9.73  (0.03; 0.56 – 8.60) | 1.72 ± 5.96  (0.16; -0.74 – 4.18) | -0.26 ± 7.44  (0.86; -3.33 – 2.81) |  | 0.06 |
| **P2, mmHg** | | | | | |
| -Baseline | 124.84 ± 10.95 | 119.76 ± 11.7 | 120.12 ± 13.65 | 0.01  Similar:  -neb/ivb |  |
| -End | 119.36 ± 10.91 | 117.58 ± 11.02 | 119.38 ± 11.2 |  |  |
| - Difference from baseline to end (P-value; 95% CI) | 5.48 ± 9.97  (0.01; 1.36 – 9.60) | 2.18 ± 6.38  (0.10; -0.45 – 4.81) | 0.74 ± 9.05  (0.69; -3.00 – 4.48) |  | 0.09 |
| **Cf-PWV, m/s** | | | | | |
| -Baseline | 10.08 ± 2.99 | 9.69 ± 2.54 | 9.57 ± 2.72 | 0.05  Similar:  -ate/neb  -neb/ivb |  |
| -End | 9.7 ± 2.42 | 9.68 ± 2.92 | 9.49 ± 2.63 |  |  |
| - Difference from baseline to end (P-value; 95% CI) | 0.38 ± 1.34  (0.17; -0.17 – 0.93) | 0.01 ± 0.89  (0.95; -0.36 – 0.38) | 0.08 ± 0.82  (0.63; -0.26 – 0.42) |  | 0.24 |

Values are presented as mean ± SD. P1 indicates pressure at the first systolic peak of the central waveform; P2, pressure at the second systolic peak of the central waveform; cf-PWV, carotid-femoral pulse wave velocity; neb, nebivolol; ivb, ivabradine; and ate, atenolol.
